# Inadequate prenatal care and its association with adverse pregnancy outcomes: A comparison of indices

**DOI:** 10.1186/1471-2393-8-15

**Published:** 2008-05-01

**Authors:** Maureen I Heaman, Christine V Newburn-Cook, Chris G Green, Lawrence J Elliott, Michael E Helewa

**Affiliations:** 1 Faculty of Nursing, Room 217 Helen Glass Centre for Nursing, University of Manitoba, Winnipeg, Manitoba, R3T 2N2 Canada; 2 Department of Obstetrics, Gynecology, and Reproductive Sciences, Faculty of Medicine, University of Manitoba, Winnipeg, Manitoba, Canada; 3 Faculty of Nursing, University of Alberta, Edmonton, Alberta, Canada; 4 Manitoba Agriculture, Food and Rural Initiatives, Winnipeg, Manitoba, Canada; 5 Department of Community Health Sciences, Faculty of Medicine, University of Manitoba, Winnipeg, Manitoba, Canada; 6 Department of Medical Microbiology, Faculty of Medicine, University of Manitoba, Winnipeg, Manitoba, Canada

## Abstract

**Background:**

The objectives of this study were to determine rates of prenatal care utilization in Winnipeg, Manitoba, Canada from 1991 to 2000; to compare two indices of prenatal care utilization in identifying the proportion of the population receiving inadequate prenatal care; to determine the association between inadequate prenatal care and adverse pregnancy outcomes (preterm birth, low birth weight [LBW], and small-for-gestational age [SGA]), using each of the indices; and, to assess whether or not, and to what extent, gestational age modifies this association.

**Methods:**

We conducted a population-based study of women having a hospital-based singleton live birth from 1991 to 2000 (N = 80,989). Data sources consisted of a linked mother-baby database and a physician claims file maintained by Manitoba Health. Rates of inadequate prenatal care were calculated using two indices, the R-GINDEX and the APNCU. Logistic regression analysis was used to determine the association between inadequate prenatal care and adverse pregnancy outcomes. Stratified analysis was then used to determine whether the association between inadequate prenatal care and LBW or SGA differed by gestational age.

**Results:**

Rates of inadequate/no prenatal care ranged from 8.3% using APNCU to 8.9% using R-GINDEX. The association between inadequate prenatal care and preterm birth and LBW varied depending on the index used, with adjusted odds ratios (AOR) ranging from 1.0 to 1.3. In contrast, both indices revealed the same strength of association of inadequate prenatal care with SGA (AOR 1.4). Both indices demonstrated heterogeneity (non-uniformity) across gestational age strata, indicating the presence of effect modification by gestational age.

**Conclusion:**

Selection of a prenatal care utilization index requires careful consideration of its methodological underpinnings and limitations. The two indices compared in this study revealed different patterns of utilization of prenatal care, and should not be used interchangeably. Use of these indices to study the association between utilization of prenatal care and pregnancy outcomes affected by the duration of pregnancy should be approached cautiously.

## Background

Prenatal care (PNC) is a frequently used health service that has the potential to reduce the incidence of perinatal morbidity and mortality by treating medical conditions, identifying and reducing potential risks, and helping women to address behavioral factors that contribute to poor outcomes[1]. Prenatal care is more likely to be effective if women begin receiving care in the first trimester of pregnancy and continue to receive care throughout pregnancy, according to accepted standards of periodicity [1]. The Society of Obstetricians and Gynecologists of Canada (SOGC) recommends that women receive PNC visits every 4 to 6 weeks in early pregnancy, every 2 to 3 weeks after 30 weeks' gestation, and every 1 to 2 weeks after 36 weeks' gestation [2], whereas the American Academy of Pediatrics (AAP) and American College of Obstetricians and Gynecologists (ACOG) recommend that a woman with an uncomplicated pregnancy be examined every 4 weeks for the first 28 weeks of pregnancy, every 2 to 3 weeks until 36 weeks gestation, and weekly thereafter[3]. Accurate measurement of PNC utilization is critical in monitoring trends and assessing the relationship between prenatal care services and pregnancy outcomes [4]. At least four indices have been developed to measure utilization of PNC [5-8], each of which uses the month that care begins and the total number of visits adjusted relative to gestational age at delivery, to assign women to categories such as "inadequate", "intermediate", "adequate", and "intensive" PNC. However, use of the Kessner index [5] and the graduated index of PNC utilization (GINDEX)[7] have largely been abandoned because the restricted nine-visit coding limitation of these indices inaccurately classifies the PNC utilization of term and post-term pregnancies [8,9]. The Adequacy of Prenatal Care Utilization (APNCU) index [6] and the revised GINDEX (R-GINDEX) [8] are both currently used to measure utilization of PNC.

Several studies using the PNC utilization indices have demonstrated an association between inadequate PNC and preterm birth or low birth weight (LBW) [10-16]. However, some investigators have questioned the use of these indices in determining an association with outcomes that are highly influenced by gestational age, such as preterm birth and LBW [9,17]. Interestingly, the outcome of small for gestational age (SGA) infants has been explored in only a few studies [17-19], even though this outcome by definition is adjusted for gestational age [17].

Several studies in the United States have reported on rates of inadequate PNC, while other studies have compared two or more of the indices in monitoring utilization of PNC [4,6,8,20]. In Canada, there are no national data on utilization of PNC [21], and only a few studies have examined rates of inadequate PNC. One study estimated that about 8.0 to 9.0% of pregnant women in Winnipeg, Manitoba received inadequate PNC in 1987–88 [22], while rates ranged from 4.4 to 10.6% in 1987–88 in a British Columbia study [23]. No studies have compared the PNC utilization indices in a Canadian context. Therefore, the objectives of this study were:

• To determine rates of PNC utilization in Winnipeg, Manitoba from 1991 to 2000;

• To compare the two most commonly used indices of PNC utilization in identifying the proportion of the population receiving inadequate PNC;

• To determine the association between inadequate PNC and preterm birth, low birth weight (LBW), and small-for-gestational age (SGA), using each of these indices; and

• To assess whether or not, and to what extent, gestational age modifies the association between inadequate PNC and LBW or SGA.

## Methods

We conducted a population-based cohort study of women having a hospital-based singleton live birth in Winnipeg, Manitoba over a ten-year period, from 1991 to 2000. Winnipeg is the capital city of the province of Manitoba, and had a population of 618,477 residents in 1996 [24]. Data sources for this study consisted of a linked mother-baby database constructed from hospital discharge abstract data and a physician claims file maintained by Manitoba Health. The project received approvals from the University of Manitoba Research Ethics Board and the Health Information and Privacy Committee of Manitoba Health.

There were 83,101 births to women residing in the city of Winnipeg from 1991 to 2000. After eliminating cases with missing or out-of-range gestational age (< 18 weeks or > 45 weeks), missing parity, maternal age less than 12 years, stillbirths or multiple births, and birth weight <400 grams but gestation >22 weeks, the final sample consisted of 80,989 births. Several mothers (37.6%) gave birth to more than one child during the ten years, and all these births were included in the analyses.

We combined two sources of data in order to estimate the number and timing of prenatal visits. We first recorded the gestational age at first visit and the total number of prenatal visits from the linked mother-baby database. These data were abstracted from the prenatal record as part of the hospital discharge abstract data system. However, several limitations of these data have been documented, including a high percent of missing information and an underestimate of timing of the first prenatal visit and total number of visits [25]. We therefore supplemented the information with data from the physician claims database. For women who received care from physicians billing on a fee-for-service basis, we determined the number of episodes of care by recording office visit tariff codes that were linked to an ICD-9-CM code indicating pregnancy (ICD-9-CM codes 640–648, 650–659, 660–669, V22, V23), and any consultation visits linked to a physician code for obstetrician/gynecologist and an ICD-9-CM code indicating pregnancy. In addition, because many physicians billed for PNC using a global tariff during the time frame of this study, direct billing of in-office or laboratory diagnostic tests was used as a surrogate measure of a PNC visit, adapted from a method previously used and validated by Mustard [25]. Finally, we determined the total number of prenatal visits by using whichever estimate of the number of visits was greater, and whichever estimate of gestational age at first visit was earlier, based on these two methods. Gestational age at delivery was determined from the newborn record. These three variables were then used to calculate the following indices of PNC utilization:

1. The Adequacy of Prenatal Care Utilization (APNCU) index, proposed by Kotelchuck[6], is comprised of two parts: the month in which PNC is initiated and the number of visits from initiation of care until delivery. Inadequate utilization is defined as either starting PNC after the 4^th ^month of pregnancy or receiving less than 50% of expected visits based on the schedule of PNC visits recommended by ACOG [3,26]. Intermediate care is care begun by month 4 and with between 50–79% of expected visits received; adequate care is that begun by month 4 and with 80–109% of expected visits received; intensive (adequate plus) care is begun by month 4 and with 110% or more of expected visits received.

2. The revised GINDEX (R-GINDEX), proposed by Alexander and Kotelchuck [8], has six categories of care: "no care," "inadequate," "intermediate," "adequate," "intensive," and "missing." The R-GINDEX is based on the full ACOG recommendation, rather than the flawed Kessner index coding strategy of a 9-visit limit. For example, at 40 weeks gestation, a woman who began prenatal care in the first 3 months and received between 13 to 16 visits would be categorized as having adequate care, whereas a woman who began care between 1 to 6 months of pregnancy and had less than 8 visits would be categorized as having inadequate care. The intensive care category includes women who have an unexpectedly large number of PNC visits, which may indicate potential morbidity or complications. Women whose number of visits is approximately one standard deviation above the mean number of visits for each trimester of initiation and gestational age at delivery are labeled as intensive care users [4].

Algorithms for calculating both of these indices have been published [7,8]. Once the two indices were calculated, we then compared the proportion of cases assigned to each category by the indices from 1991 to 2000.

Differences in rates of inadequate/no PNC by maternal age and parity were calculated. SGA births (birth weight less than 10^th ^percentile for gestational age) were determined using a population-based Canadian reference [27]. Logistic regression analysis with generalized estimating equation parameter estimates (GEE) was used to determine the association between inadequate/no PNC, using both indices, and birth outcomes (preterm birth, LBW, and SGA) after controlling for maternal age and parity, and adjusting for more than one birth to the same mother (i.e., within-mother dependency). Inadequate/no prenatal care was compared to the reference group of all other types of care (intermediate, adequate and intensive).

A stratified analysis was conducted to determine whether the association between inadequate/no PNC and LBW or SGA differed by gestational age. The Breslow-Day test of homogeneity was used to test the null hypothesis that the effect measure was uniform across strata [28].

## Results

Table 1 compares the proportion of women assigned to various categories of PNC utilization based on the two indices. Only a small proportion of women (n = 293; 0.4%) received no care during the 10 years. The overall proportion of women assigned to the inadequate category varied slightly among the two indices, ranging from 7.9% for APNCU to 8.5% for R-GINDEX. The APNCU assigned a much higher proportion of women to the "intensive" care category (31.4%) than did the R-GINDEX (12.6%). Because of the small proportion of women receiving no care, the categories of inadequate and no care were combined for most of the remaining analyses. Table 2 summarizes the proportion of women with inadequate/no care by maternal age and parity. Women aged less than 20 years had the highest rates of inadequate/no care (ranging from 21.1% to 21.6%) while women aged 35 years and older had the lowest rates. Women with the highest level of parity (4 or more births) had the highest rates of inadequate/no PNC (ranging from 24.3% to 26.4%) while women having their first birth had the lowest rates.

**Table 1:** Rates of prenatal care utilization in Winnipeg, 1991–2000: A comparison of two indices

Index	Categories	1991	1992	1993	1994	1995	1996	1997	1998	1999	2000	Overall
		9093	8966	8725	8726	8440	8039	7478	7210	7188	7124	80989*
		(%)	(%)	(%)	(%)	(%)	(%)	(%)	(%)	(%)	(%)	(%)
APNCU Index												
	Intensive	26.6	32.9	33.1	31.9	32.2	32.1	31.7	31.3	28.4	33.6	31.4
	Adequate	40.3	39.6	40.8	41.9	39.7	38.5	38.6	37.4	36.2	40.9	39.5
	Intermediate	24.1	19.0	17.7	18.4	20.3	21.6	22.2	23.1	26.0	17.5	20.9
	Inadequate	8.6	8.0	7.9	7.4	7.5	7.6	7.3	7.9	8.9	7.8	7.9
	No prenatal care	0.4	0.4	0.5	0.4	0.3	0.3	0.2	0.3	0.5	0.3	0.4
R-GINDEX												
	Intensive	10.8	13.5	13.6	12.8	12.4	13.2	12.3	12.8	11.0	13.4	12.6
	Adequate	37.2	43.1	44.0	44.0	42.8	40.3	41.3	39.4	38.0	42.3	41.3
	Intermediate	42.3	34.8	33.8	35.3	36.6	37.9	38.1	38.0	39.7	36.1	37.2
	Inadequate	9.2	8.2	8.2	7.5	7.9	8.3	8.2	9.5	10.8	7.9	8.5
	No prenatal care	0.4	0.4	0.5	0.4	0.3	0.3	0.2	0.3	0.5	0.3	0.4

*Number of singleton live births per year and overall

**Table 2:** Inadequate/no prenatal care utilization by maternal age and parity, Winnipeg, 1991–2000: A comparison of two prenatal care utilization indices

Index	Maternal Age (years)			Parity		
	
	<20	20–34	35 +	1^st ^birth	2^nd^-3^rd ^birth	≥4^th ^birth
APNCU Index						
% inadequate/no care	21.1	7.5	4.3	6.2	7.5	24.3
R-GINDEX						
% inadequate/no care	21.6	8.2	4.8	6.2	8.4	26.4

The proportion of preterm births and LBW by category of PNC varied among the two indices (Table 3), with highest rates in the no PNC group (15.0%). Using the APNCU, the rate of preterm birth in the inadequate care group (7.2%) was approximately double that of the adequate care group (3.5%). However, this was not true of the R-GINDEX, where the rate of preterm birth in the inadequate care group (6.1%) was lower than that in the adequate care group (8.8%). Using the APNCU, a high rate of preterm birth (10.8%) was also found in the intensive care category. The rate of SGA was more consistent across the indices, with the highest rate (18.8%) in the no care category and similar rates in the inadequate care category for the APNCU (13.0%) and the R-GINDEX (12.8%).

**Table 3:** Comparison of two prenatal care utilization indices: Percent of singleton live births assigned to each category and percent of preterm, low birth weight, and small-for-gestational age births among live births in each category, Winnipeg, 1991–2000

Index	Categories	Singleton live births	Preterm Births	Small-for-gestational-age (SGA)	Low birth weight (LBW)
		
		No. (%)	No. (%)	No. (%)	No. (%)
APNCU Index					
	Intensive	25 394 (31.4)	2 725 (10.7)	2 379 (9.4)	1 920 (7.6)
	Adequate	31 982 (39.5)	1 130 (3.5)	2 981 (9.3)	1 011 (3.2)
	Intermediate	16 937 (20.9)	463 (2.7)	1 717 (10.1)	494 (2.9)
	Inadequate	6 383 (7.9)	458 (7.2)	832 (13.0)	389 (6.1)
	No prenatal care	293 (0.4)	44 (15.0)	55 (18.8)	44 (15.0)
R-GINDEX					
	Intensive	10 192 (12.6)	326 (3.2)	929 (9.1)	255 (2.5)
	Adequate	33 453 (41.3)	2 951 (8.8)	3 134 (9.4)	2 201 (6.6)
	Intermediate	30 139 (37.2)	1 077 (3.6)	2 960 (9.8)	993 (3.3)
	Inadequate	6 912 (8.5)	422 (6.1)	886 (12.8)	365 (5.3)
	No prenatal care	293 (0.4)	44 (15.0)	55 (18.8)	44 (15.0)

Table 4 reports the unadjusted and adjusted odds ratios (AOR) for the association between inadequate/no PNC care and pregnancy outcomes. Using the APNCU, the likelihood of preterm birth and LBW associated with inadequate or no PNC was significantly increased by 20% and 30% respectively (AOR 1.2 and 1.3). Using the R-GINDEX, there was no association between inadequate/no care and preterm birth (AOR 1.0) and a weak association with LBW (AOR 1.1). In contrast, both indices yielded the same result for the outcome of SGA, with the likelihood of SGA being significantly increased by 40% (AOR 1.40) among women with inadequate/no prenatal care.

**Table 4:** a – Odds ratios (OR) and 95% confidence intervals (CI) for association of inadequate/no prenatal care with preterm birth (< 37 completed weeks gestation)* b – Odds ratios (OR) and 95% confidence intervals (CI) for association of inadequate/no prenatal care with low birth weight (<2500 grams)* c – Odds ratios (OR) and 95% confidence intervals (CI) for association of inadequate/no prenatal care with small-for-gestational age (birth weight < 10^th^ percentile for gestational age)*

a – Odds ratios (OR) and 95% confidence intervals (CI) for association of inadequate/no prenatal care with preterm birth (< 37 completed weeks gestation)*				
Index	Unadjusted OR	95% CI	Adjusted OR**	95% CI
APNCU Index	1.3	1.2 – 1.5	1.2	1.1 – 1.3
R-GINDEX	1.1	1.0 – 1.2	1.0	0.9 – 1.1

b – Odds ratios (OR) and 95% confidence intervals (CI) for association of inadequate/no prenatal care with low birth weight (<2500 grams)*				

Index	Unadjusted OR	95% CI	Adjusted OR**	95% CI

APNCU Index	1.4	1.3 – 1.6	1.3	1.2 – 1.5
R-GINDEX	1.2	1.1 – 1.4	1.1	1.0 – 1.3

c – Odds ratios (OR) and 95% confidence intervals (CI) for association of inadequate/no prenatal care with small-for-gestational age (birth weight < 10^th^ percentile for gestational age)*				

Index	Unadjusted OR	95% CI	Adjusted OR**	95% CI

APNCU Index	1.5	1.4 – 1.6	1.4	1.3 – 1.5
R-GINDEX	1.4	1.3 – 1.5	1.4	1.3 – 1.5

* Inadequate/no prenatal care was compared to the reference group of all other types of care (intermediate, adequate and intensive).

**Controlling for maternal age and parity, and adjusting for within-mother dependency. Maternal age was studied using categories of <20 years, 20–24 years, 25–29 years, 30–34 years, and 35+ years, with 35+ years as the reference group. Parity was studied using categories of first birth, 2^nd^-3^rd ^birth and 4 or more births, with first birth as the reference group.

The stratified analyses of the association between inadequate/no PNC and SGA and LBW are reported in Tables 5 and 6 respectively. The odds ratios varied widely across gestational age categories, with the association between inadequate/no PNC and both LBW and SGA increasing towards term. The Breslow-Day test of homogeneity was significant for the association between APNCU and both SGA and LBW, and between R-GINDEX and SGA, indicating the presence of heterogeneity (non-uniformity) across strata. The Breslow-Day test for the association between R-GINDEX and LBW suggested a trend towards significance (p = 0.06) and the possibility of non-uniformity across strata.

**Table 5:** Odds ratios (OR) for association of inadequate/no prenatal care with SGA by gestational age category: Comparison of two prenatal care utilization indices.

Gestation	Total n	SGA n	APNCU OR	R-GINDEX OR
<27 weeks	263	57	ne*	ne*
27–28	177	22	0.82	1.84
29–30	186	20	1.31	0.92
31–32	403	38	0.23	0.25
33–34	865	78	0.27	0.77
35–36	2 926	321	0.81	0.89
37–38	16 024	1 352	1.40	1.38
39–40	45 115	4 515	1.48	1.47
41–42	14 964	1 552	1.80	1.64
43–44	66	9	2.71	1.73

Breslow-Day Test			p = 0.0001	p = 0.0154

*ne = not estimable.

**Table 6:** Odds ratios (OR) for association of inadequate/no prenatal care with LBW (<2500 grams) by gestational age category: Comparison of two prenatal care utilization indices.

Gestation	Total n	LBW n	APNCU OR	R-GINDEX OR
<27 weeks	263	262	ne*	ne*
27–28	177	172	ne*	ne*
29–30	186	177	0.28	0.43
31–32	403	380	0.72	1.03
33–34	865	646	0.85	1.02
35–36	2 926	954	1.19	1.22
37–38	16 024	762	1.48	1.36
39–40	45 115	445	1.64	1.45
41–42	14 964	60	2.85	3.45
43–44	66	0	ne*	ne*

Breslow-Day Test			p = 0.0159	p = 0.0624

*ne = not estimable.

## Discussion

The proportion of cases assigned to PNC utilization categories varied using the two different indices. This finding is similar to the conclusions of Alexander and Kotelchuck [8] and Kogan et al [4]. Because the indices use different algorithms to define categories of PNC utilization, they yield different patterns of PNC use in a population and should not be used interchangeably [8]. This emphasizes the need to use caution in comparing results across studies that use different indices. The proportion of women assigned to each PNC utilization category remained fairly stable over the 10 years for both indices, although it is noteworthy that the number of births steadily declined over the 10 years, from 9,093 births in 1991 to 7,124 births in 2000. This declining birth rate is consistent with that reported in a provincial surveillance report for Manitoba, 1989–1998 [29].

The rates of inadequate and no PNC utilization among women in Winnipeg are lower than those reported in the United States, likely as a result of universal health care for residents of Manitoba. About 1.5 to 2.0% of pregnant women in the United States do not receive any PNC [30], compared to 0.4% among Winnipeg women in this study. Using the APNCU index, the rate of inadequate/no PNC in the United States declined from 12.8% in 1995 to 11.7% in 1999 [31], but these rates are still considerably higher than the 8.3% of women in Winnipeg who received inadequate/no PNC from 1991 to 2000 based on the APNCU index.

The association between inadequate/no prenatal care and LBW varied using the two indices, with AOR ranging from 1.1 using the R-GINDEX to 1.3 using the APNCU. Caution needs to be used in interpreting these results because our analyses also confirmed the presence of effect modification by gestational age for both indices, with the association between inadequate/no care and LBW becoming stronger as gestational age increased. Koroukian and Rimm state, "Although adjusting the number of prenatal visits for gestational length is clearly important to assess the adequacy of prenatal care utilization, we must be mindful of the bias introduced by its use in an Index, because the gestational length is itself a birth outcome that is so strongly correlated with birth weight"[17]. Alexander and Kotelchuck suggest the use of different analytical approaches to examine the relationship between prenatal care use and birth outcomes, such as gestational age-specific, life table, survival, and two stage least squares analyses, to help control for the influence of gestational age [9]. There is growing concern that the strength of the relation between PNC and LBW and preterm birth may be far less than previously assumed [32,33]. This lack of association may result because PNC, in its present form, has limited ability to reduce the proportion of LBW and preterm births; however, some evidence suggests that PNC may make a difference in term LBW rates [32,34]. This lack of association might also explain why efforts to prevent preterm birth and LBW through increased access to PNC have shown little benefit [35,36].

Of the three adverse pregnancy outcomes selected for this study, SGA may be the preferable outcome to study because by definition it adjusts for gestational age. However, our findings still showed some degree of effect modification by gestational age when studying the association between inadequate/no PNC and SGA, so the results should be interpreted with caution. After controlling for maternal age, parity, and within-mother dependency, women receiving inadequate/no PNC were up to 40% more likely to have a SGA birth compared to women receiving other categories of care. This result is consistent with the findings of a New Zealand study that less frequent attendance at PNC was associated with SGA [18]. The reason for the observed association between inadequate PNC and SGA births is not fully understood. However, it is likely that women who do not receive adequate PNC are less likely to receive appropriate treatment or preventive care. SGA births are associated with several potentially modifiable risk factors, such as low pre-pregnancy weight, low gestational weight gain, cigarette smoking, and recreational drug use [37,38]. Several of these risk factors may be mitigated or prevented with quality PNC.

As with most research, this study has limitations. First, administrative data are prone to a certain degree of coding errors and incomplete data, which may be random or contain systematic biases. The number and timing of PNC visits was estimated from hospital discharge abstracts and physician claims files, and the accuracy of our estimates may be affected by several factors, such as missing PNC records or receipt of PNC from non-physician providers. We were unable to differentiate missing data from no care using this approach. As well, inaccurate ascertainment of gestational age may affect assignment to a PNC utilization category or determination of a preterm or SGA birth. We compared the rate of adverse birth outcomes among women with inadequate/no PNC to the remainder of the population. However, Kotelchuck suggests there is a U-shaped relationship between PNC and birth outcomes, in which women with both fewer and greater number of visits than expected are at higher risks of having poorer birth outcomes [39], so perhaps limiting the reference group to women with adequate care should be considered in future research. Our analysis was limited to singleton live births; therefore, multiple births were not represented. In addition, a limitation of both PNC utilization indices is that they only reflect the quantity of PNC; they indicate nothing about the spacing of visits or the content, clinical adequacy, or quality of PNC [8]. These indices are based on the ACOG recommendations for number of visits for low risk pregnant women; the effectiveness of this standard has not been assessed through rigorous scientific testing, nor has adequacy of care for women with high risk pregnancies been operationalized [9]. Last, selection bias is a major difficulty in assessing the impact of PNC on pregnancy outcomes, in that women who receive adequate PNC may be more likely to experience better pregnancy outcomes because of other characteristics which have independent influences on pregnancy outcomes [38,40]. We were unable to control for maternal characteristics such as socioeconomic status, race/ethnicity, intendedness of pregnancy or health behaviors because information on these variables are not recorded in the databases.

## Conclusion

The rates of no PNC and inadequate PNC are lower for women giving birth in Winnipeg, Canada, compared to rates reported for women in the U.S. The two indices compared in this study revealed different utilization patterns and resulted in varying degrees of association of inadequate PNC with adverse pregnancy outcomes. Selection of a PNC utilization index for research or program evaluation requires careful consideration of the methodological underpinnings and limitations of the chosen index [8]. Although these indices remain useful for studying trends in PNC utilization or evaluating the effectiveness of programs to enhance access to care, we concur with other investigators that use of these indices to study the association between utilization of PNC and birth outcomes affected by the duration of gestation should be approached with caution due to effect modification by gestational age [8,17]. In addition, "more refined future indices should incorporate parameters that reflect the qualitative aspects of PNC in addition to measuring number of visits" [8]. Future research should go beyond simply counting the number of visits and focus on studying the relationship between quality and content of PNC and pregnancy outcomes. There is a pressing need to develop a valid and reliable instrument to measure quality of PNC.

## Competing interests

The authors declare that they have no competing interests.

## Authors' contributions

MIH conceived of the study and supervised all aspects of its implementation. All authors contributed to conceptualizing ideas and designing the study, provided input regarding analysis of the data, interpreted findings, and reviewed drafts of the manuscript. All authors read and approved the final manuscript.

## Pre-publication history

The pre-publication history for this paper can be accessed here:


